# Common variable immunodeficiency unmasked by treatment of immune thrombocytopenic purpura with Rituximab

**DOI:** 10.1186/2052-1839-13-4

**Published:** 2013-04-11

**Authors:** Trine H Mogensen, Jens Magnus Bernth-Jensen, Charlotte C Petersen, Mikkel S Petersen, Charlotte Nyvold, Karsten H Gadegaard, Marianne Hokland, Peter Hokland, Carsten S Larsen

**Affiliations:** 1 Department of Infectious Diseases, Aarhus University Hospital, Skejby Brendstrupgaardsvej, DK-8200, Aarhus N, Denmark; 2 International Center for Immunodeficiency Diseases, Aarhus University Hospital, Skejby, Aarhus, Denmark; 3 Department of Clinical Immunology, Aarhus University Hospital, Skejby, Aarhus, Denmark; 4 Department of Biomedicine, Aarhus University, Aarhus, Denmark; 5 Department of Haematology, Aarhus University Hospital, Aarhus, Denmark; 6 Department of Anaesthesiology, Aarhus University Hospital, Aarhus, Denmark

**Keywords:** Hypogammaglobulinemia, Common variable immunodeficiency, Immune thrombocytopenic purpura, Rituximab

## Abstract

**Background:**

Hypogammaglobulinemia may be part of several different immunological or malignant conditions, and its origin is not always obvious. Furthermore, although autoimmune cytopenias are known to be associated with common variable immunodeficiency (CVID) and even may precede signs of immunodeficiency, this is not always recognized. Despite novel insight into the molecular immunology of common variable immunodeficiency, several areas of uncertainty remain. In addition, the full spectrum of immunological effects of the B cell depleting anti-CD20 antibody Rituximab has not been fully explored. To our knowledge this is the first report of development of CVID in a patient with normal immunoglobulin prior to Rituximab treatment.

**Case presentation:**

Here we describe the highly unusual clinical presentation of a 34-year old Caucasian male with treatment refractory immune thrombocytopenic purpura and persistent lymphadenopathy, who was splenectomized and received multiple courses of high-dose corticosteroid before treatment with Rituximab resulted in a sustained response. However, in the setting of severe pneumococcal meningitis, hypogammaglobulinemia was diagnosed. An extensive immunological investigation was performed in order to characterize his immune status, and to distinguish between a primary immunodeficiency and a side effect of Rituximab treatment. We provide an extensive presentation and discussion of the literature on the basic immunology of CVID, the mechanism of action of Rituximab, and the immunopathogenesis of hypogammaglobulinemia observed in this patient.

**Conclusions:**

We suggest that CVID should be ruled out in any patient with immune cytopenias in order to avoid diagnostic delay. Likewise, we stress the importance of monitoring immunoglobulin levels before, during, and after Rituximab therapy to identify patients with hypogammaglobulinemia to ensure initiation of immunoglobulin replacement therapy in order to avoid life-threatening invasive bacterial infections. Recent reports indicate that Rituximab is not contra-indicated for the treatment of CVID-associated thrombocytopenia, however concomitant immunoglobulin substitution therapy is of fundamental importance to minimize the risk of infections. Therefore, lessons can be learned from this case report by clinicians caring for patients with immunodeficiencies, haematological diseases or other autoimmune disorders, particularly, when Rituximab treatment may be considered.

## Background

Humoral immunity is dependent upon a full repertoire of mature B-lymphocytes capable of adequately mounting a primary and secondary immune response. Lack of this will invariably lead to severe bacterial infections as can be evaluated by the disease spectrum in inborn and acquired immunodeficiencies. Regarding the former, common variable immunodeficiency (CVID) is a heterogeneous entity characterized by varying degrees of hypogammaglobulinemia and recurrent bacterial infections. Patients with CVID also experience an increased risk of granulomatous and autoimmune manifestations as well as malignancy [1,2]. Autoimmune manifestations, among which Immune thrombocytopenic purpura (ITP) and autoimmune hemolytic anemia (AIHA) are the most frequent, occur in as many as 20–40% of CVID patients and often precede symptoms of immune deficiency [3]. Cytopenias, and particularly ITP, may therefore be the initial presentation of CVID, and recognizing a possible underlying immunodeficiency may have important implications for the choice of treatment.

Acquired hypogammaglobulinaemia is typically associated with malignant disorders in the B-cell lineage such as B- cell chronic lymphocytic leukemia (B-CLL), B-cell derived non-Hodgkin’s lymphomas (NHL) or multiple myeloma. Here, progressive disease in combination with cytoreduction leads to secondary hypogammaglobulinemia and ensuing infections. Finally, immunosuppressive therapy targeted at B-cells, e.g. in autoimmune disorders can lead to similar clinical presentations, admittedly often to a lesser extent.

ITP is a condition, in which the challenge is to alleviate thrombocytopenia without inducing severe immunosuppression. Thus, while most cases can be easily treated and will not recur, others will relapse without any apparent trigger and will turn out to be increasingly difficult to treat. The current management of ITP usually consists of an initial course of corticosteroids. Should the condition recur and corticosteroid therapy be either not effective or not feasible, high-dose immunoglobulins, perhaps followed by splenectomy used to be accepted standard therapy. However, given that a number of patients refractory to standard immunosuppressive treatment have been successfully treated with Rituximab, an anti-CD20 chimeric antibody depleting CD20-expressing B cells in lymphoid tissues and in the circulation, this regimen is rapidly gaining acceptance as the treatment of choice at relapse of ITP [4]. It is known that repeated courses of Rituximab in NHL patients generally do not lead to hypogammaglubulinaemia, nor increased frequency of infections. However, the extent and duration of B cell depletion and effects on immunoglobulin levels in ITP patients, especially those pretreated with corticosteroids, high-dose Ig and splenectomy, are not well described. Furthermore, growing evidence suggest that Rituximab not only depletes cells within the B cell compartment, but also may influence T cell immunity [4]. Therefore the long-term effects of Rituximab treatment, in particular with respect to the immune competence of the host, need to be better explored.

Here we describe an ITP patient, in whom Rituximab was instituted some 24 years after onset, and in whom the prior therapy might have predisposed him to the persistent hypogammaglobulinemia observed, which resulted in a severe infection requiring immunoglobulin replacement therapy.

We review the literature on CVID, ITP, and Rituximab and discuss whether this patient’s hypogammaglobulinemia was due to underlying CVID, or alternatively was induced directly by Rituximab treatment.

## Case presentation

A 34-year old man was referred to the International Center of Immunodeficiency Diseases (ICID) in January 2011 with hypogammaglobulinemia diagnosed in the context of severe pneumococcal meningitis, for which he had been hospitalized in the intensive care unit the previous month. The patient, who was splenectomized due to ITP, had experienced a very rapid and aggressive clinical course with invasive disease despite the use of oral penicillin prior to admission. In the intensive care unit he had been treated with standard antibiotic therapy, including intravenous penicillin and ceftriaxone as well as dexamethasone. He had made an uneventful recovery without neurological deficits. However, persistently reduced levels of serum IgG of 2.6 g/L (6.1 g/L - 14.9 g/L) and IgA of 0.14 g/L (0.80 g/L – 3.90 g/L) but normal IgM of 0.73 g/L (0.39 g/L – 2.08 g/L) remained.

### Past medical history

The patient had been diagnosed with ITP at 11 years of age in 1987. At that time, he had severe thrombocytopenia (3 × 10^9^ cells/L), modest leukopenia (2.2 × 10^9^ cells/L) and lymphadenopathy. Peripheral blood smear, bone marrow biopsy, and lymph node biopsy all displayed normal histology. According to his pediatric medical file, he reported no increased frequency of bacterial infections during childhood, and neither relatives nor siblings had any known immunodeficiencies or autoimmune diseases. He was treated with thrombocyte infusions and intravenous immunoglobulin (0.4 g/kg) followed by a three-week course of corticosteroid (2 mg/kg) with a resultant rapid increase in thrombocyte count. Unfortunately, immunoglobulin levels were not measured at the time. Due to several relapses of severe thrombocytopenia with each tapering of corticosteroid treatment, he was subsequently splenectomized in 1988. Hepato-splenomegaly was observed during the operation, and pathological evaluation of the spleen displayed hyperplasia without malignancy. In 2001, several accessory spleens were scintigraphically demonstrated but not removed.

During the following years, he was followed at the University Hospital Department of Hematology, and several episodes of severe thrombocytopenia accompanied by bruises and petecchiae occurred, each time responding immediately, but transiently, to high-dose corticosteroid treatment. Lymph node biopsy due to persistent lymphadenopathy again revealed benign hyperplasia. Due to lack of an acceptable response to repeated courses of high-dose corticosteroid (100 mg daily), the patient was treated in January 2009 with four doses of Rituximab (375 mg/m^2^ iv.) one week apart. He had an excellent response with normalization of thrombocyte counts and a lasting response, so far for 36 months. Based on the stable platelets and the fact that the patient was a medical doctor, his contacts with the Hematology Department were restricted to e-mails based on platelet counts performed twice a year.

### Hypogammaglobulinemia, the infectious episode, and its elucidation

After a vacation in Mallorca spent sailing in December 2010 the patient returned home with an upper respiratory tract infection as well as diarrhoea and vomiting. The patient developed high fevers and confusion and was admitted to the Department of Infectious Diseases, where pneumococcal meningitis was diagnosed. Within a few hours the patient became unconscious necessitating transfer to the Intensive Care Unit on mechanical ventilation, where he received ten days of antibiotic treatment and made a successful recovery. At presentation in the ICID with hypogammaglobulinemia in January 2011, it turned out that the patient had been suffering from several infections during the past two years, since Rituximab treatment in January 2009. He reported frequent episodes of upper airway infections, (although no sinusitis or pneumonia) and gastrointestinal symptoms, including almost daily discomfort and diarrhoea. According to his pediatric medical file, he had received appropriate vaccination before the splenectomy, and also reported having followed up correctly on pneumococcal antibody status and re-vaccination as prescribed.

**Figure 1:**
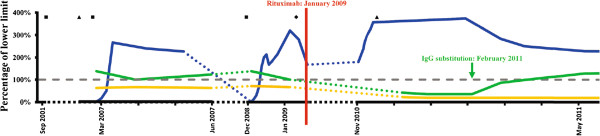
**Laboratory parameters and treatment over time.** Plasma IgG (green), plasma IgA (yellow), and thrombocyte count (blue) are illustrated as percentages of reference lower limit (6.0 g/L, 0.78 g/L, and 150 × 10^^9^/L respectively). Time of Rituximab treatment (red line) and initiation of IgG substitution therapy (green arrow) are also depicted. Thrombocyte-binding IgGs identified in serum (square) or bound to the patient’s thrombocytes but undetectable in serum (diamond) are marked. Finally, time points for measurement of insufficient concentrations of pneumococcal polysaccharide-binding IgGs are marked (triangle).

An extensive immunological evaluation was performed in an attempt to distinguish between a primary immunodeficiency possibly aggravated by Rituximab treatment or, alternatively, hypogammaglobulinemia induced by Rituximab treatment. The results are summarized below and illustrated in Figure 1.

### Serum antibody levels

The patient had severely reduced serum IgG (2.6 g/L) with all subclasses affected except IgG3, and reduced IgA (0.14 g/L), whereas IgM was normal. Blood tests taken before Rituximab treatment revealed that selective IgA deficiency was present already in 2007, whereas normal levels (although only slightly above the lower limit dating back to 2001) of serum IgG had been measured at several occasions during the period between February 2007 and January 2009. In addition, low levels of isohaemagglutinins were present. Thrombocyte-specific antibodies (IgG with unknown allo-specificity) were present repeatedly in 2001, 2007, and 2008. In January 2009 just prior to Rituximab infusion membrane-bound but no soluble anti-thrombocyte antibodies were detectable, but were absent in 2010 and 2011 at several occasions. Finally, in March 2007 he had insufficient pneumococcal antibody levels following latest vaccination in February 2001. During the first evaluation in our department in 2010 he again had insufficient pneumococcal antibody levels despite vaccination in 2007 as well as in 2009 prior to Rituximab treatment. Antibody levels against difteria- and tetanus toxin were within normal limits in December 2010.

### B and T cell subsets

The number of B cells in the peripheral blood was evaluated by flow cytometry in 2001 and revealed a normal fraction of CD19/CD20+ cells without evidence of light chain restriction. At evaluation in January 2011, flow cytometry revealed a severely reduced fraction (0.1%) of isotype-switched memory B cells (CD19/20+ IgD- IgM- CD38- CD27+) (Additional file 1: Table S1). Additionally, selective reductions in marginal zone-like B cells (CD19/20+ IgD + IgM + CD38- CD27+) and absence of plasmablasts (CD19+ CD20- IgD- IgM+/− CD38+ CD27+) were observed. Within the T cell compartment only minor abnormalities were demonstrated (Additional file 1: Table S1), including a major, and possibly increased fraction of 15% of CD4- CD8- double negative T cells (normal range 4–11%) [5,6], a slightly decreased fraction of naïve cells among CD4 T cells, and a bias towards a terminally differentiated memory fraction among CD8 T cells. Consistent with this, lymphocyte proliferation analyses in response to a range of mitogens (PHA and PWM) and antigens (PPD, *C. albicans, S. aureus*, and tetanus toxoid) were all within normal limits. Histopathology and flow analysis of the bone marrow aspirate demonstrated a normal bone marrow with stimulated thrombopoiesis but without the existence of clonality or malignancy. Additional flow analyses of the bone marrow aspirate demonstrated abnormal distribution of B cells with increased fraction of CD19+ CD20- and absence of IgD- IgM- CD27+ memory B cells (Additional file 2: Figure S1).

### Other data

Complement activation, including classical-, alternative-, and lectine pathways, were all normal.

### Molecular biology analyses

By Ig gene rearrangement analysis no evidence of clonality was observed in the unfractionated B-cells or in B-cell subsets obtained by FACS. Somatic hypermutation within immunoglobulin kappa gene transcripts was not detected, although the result was uncertain due to very low numbers of B cells. Mutations associated with CVID were not demonstrated in either ICOS or TACI, and other potential mutations within BAFF-R, CD19, CD20 or CD81 were not analyzed.

Based on the results of these immunological analyses, particularly the low number of isotype- switched memory B cells, a presumptive diagnosis of CVID was made. Within CVID classification systems, the patient fulfilled the criteria for Freiburg type Ia and EUROClass group smB-Tr(high) CD21(low). In February 2011 subcutaneous immunoglobulin replacement therapy was initiated with Subcuvia 60 mL weekly and within three months of treatment his IgG level had risen to 7.6 g/L. In June 2011 flow cytometric evaluation of B and T cell subsets was performed, demonstrating a persistently significantly reduced fraction of isotype-switched memory B cells of 0.1%, although his total circulating CD19+ B lymphocyte count had risen to 1089 cells × 10^6^/L (21.3% of total lymphocytes) compared to 285 cells × 10^6^/L (12.1% of total lymphocytes) in January 2011 (Additional file 1: Table S1). Currently as of October 2012, he is doing well, does not experience increased frequency of bacterial infections receiving 60 mL subcutaneous immunoglobulin weekly (obtaining an IgG level ranging between 8 g/L and 10 g/L). Importantly, he preserves a normal thrombocyte count of 335 × 10^9^ cells/L more than three years after Rituximab treatment for ITP.

## Discussion

Here, we present the medical history of a patient with treatment refractory ITP and persistent lymphadenopathy diagnosed at the age of 11-years, who was splenectomized and received multiple courses of high-dose corticosteroid before he was eventually treated with Rituximab resulting in a sustained response with normalization of thrombocyte counts. However, in the setting of severe pneumococcal meningitis, hypogammaglobulinemia was diagnosed, and an extensive immunological investigation was performed in order to characterize the immune status of the patient, and to distinguish between a primary immunodeficiency and a side effect of Rituximab treatment.

### Common variable immunodeficiency

The hallmark of CVID is increased susceptibility to bacterial respiratory tract infections caused by reduced levels of serum immunoglobulins. CVID is defined as significantly reduced levels of IgG and decreased levels of IgA and/or IgM together with an absence of iso-haemagglutinins and/or inappropriate responses to pneumococcal vaccination [1,2]. Several different classification systems have been developed in order to divide this broad spectrum of immunodeficiencies into biologically and clinically meaningful groups predicting severity of disease, treatment response, and prognosis [7,8]. The Freiburg classification allows a division of patients into three distinct groups and emphasizes that the central characteristic of CVID is lack of switched memory B cells (IgD- IgM- CD27+) [8,9], which is associated with the development of autoimmunity, lymphoid hyperplasia, splenomegaly, and granulomatous disease [10,11] and therefore a major determinant of disease severity. Another aspect of CVID is that somatic hypermutation is impaired in B cells of about 70% of patients, thus interfering with the production of highly specific antibodies [12]. Although CVID primarily affects the B cell population and immunoglobulin synthesis, it may also influence T cell immunity. Numerous abnormalities in the T cell compartment have been reported in CVID patients, including T lymphopenia, decreased CD4 + CD45RA + T cells, a restricted T cell repertoire, accelerated T cell death, and impaired cytokine production [13]. As to the origin of CVID, a number of genetic defects, including mutations in TACI, ICOS, BAFF-R, CD19, CD20, and CD81, which are all molecules involved in differentiation, survival, and activation of B cells, have been identified. These only account for a minority of CVID cases (about 15-20%), and the molecular pathophysiology of the remaining fraction has remained largely obscure [14]. Recently, however, a genome-wide association study has added novel mutations to this list by identifying diverse causes of CVID [15]. Taken together, CVID cannot simply be characterized as a condition of impaired immune responses, but should rather be viewed as state of aberrant immunity.

### Autoimmunity in CVID

A substantial proportion of patients with CVID also experience autoimmune manifestations, ranging from 22% to 48% in different studies, which is associated with the presence of granulomatous disease and TACI mutations [3,7]. Among autoimmune conditions associated with CVID, cytopenias are particularly common, often present together with splenomegaly [7]. In different case series, ITP has been diagnosed in 7% to 20% of patients with CVID [7,16,17]. In the context of the present case it is notable that the diagnosis of ITP and/or AIHA frequently precedes that of CVID, which was indeed the case in 54% of patients in one study [16] and in 62% in another study [17]. Besides ITP and AIHA, other hematologic manifestations overrepresented among CVID patients are polyclonal lymphocytic infiltration and lymphoid malignancy, each of which is positively correlated with the serum IgM level [7]. Several hypotheses have been presented to explain the increased frequency of autoimmune manifestations in CVID. Given that both defective B cell class-switch and loss of somatic hypermutation are correlated to autoimmunity it has been suggested that such molecular alterations may explain the inability to exclude autoreactive B cell clones [13].

### Clinical use of Rituximab

Rituximab is an anti-CD20 humanized chimeric monoclonal antibody and was initially developed to treat B cell malignancies [4,18]. Following initial success, Rituximab has also been approved for the management of rheumatoid arthritis and has undergone clinical trials demonstrating the successful use in a growing number of autoimmune diseases, including systemic lupus erythematosis, ITP, pemphigus, multiple sclerosis, and ANCA-associated vasculitis [19-23], in which dysregulated B cells are involved in the pathogenesis. A systematic review on the utilization of rituximab in ITP was published in 2007, included 313 patients from a total of 19 (eligible) studies and reported an overall response rate of 62.5% (as defined by a platelet increase to >50 × 10^9^ cells/L) when given at a dose of 375 mg/m^2^ iv once weekly for 4 weeks [24]. The median duration of the response was 10.5 months [24]. More recent reports indicate that Rituximab may be more effective than various other immunosuppressants, particularly in the setting of refractory cases of ITP. Accordingly, Rituximab has been recognized as a useful drug in the second line of treatment for ITP either alone or in combination with corticosteroid, although it is not recommended as standard first-line therapy [25]. However, whereas initial response rates are high, long-term follow up data on patients treated with Rituximab are sparse. It is still under investigation, how patients should be monitored and how frequently treatment should be repeated. In general, Rituximab is considered a relatively safe drug with only modest toxicity and infectious complications [4]. Among side effects described are infusion-related reactions, serum sickness, and agranulocytosis [19,26]. Due to the depletion of the B cell-mediated arm of the immune system, infections are a major concern. In different studies, the rate of serious infections has been between 2.8% to 45% (mean 12%) and the use of Rituximab as a direct cause of death has been estimated to 7% [27]. These numbers may be difficult to interpret, since most of the patients were immunocompromised even prior to Rituximab treatment due to autoimmunity and the concomitant use of additional immunosuppressive agents. A recent multicenter retrospective study on the use of Rituximab in CVID-associated immune cytopenias reported a response rate of 85%, including 74% complete responses. However, after a mean follow-up time of 30 months after Rituximab, 10 out of the initial reponders relapsed and required re-treatment, and severe infections occurred in 24% [28].

### Mechanism of action of Rituximab

Rituximab targets CD20 expressed by human B cells and a small subset of T cells [29,30]. Within the B cell compartment, the large majority of B cells, with the exception of stem cells, pro-B cells, and plasma cells, express CD20 and therefore represent targets of Rituximab [31]. Binding to CD20 by Rituximab induces B cell depletion by complement- and antibody-mediated cytotoxicity resulting in a 90% reduction in the number of CD20+ B cells in peripheral blood, lymphoid tissue, and bone marrow [32]. The rationale behind using Rituximab in malignant and autoimmune diseases is therefore to eradicate malignant B cell clones or to prevent production of auto-antibodies by auto-reactive short-lived plasma cells derived from CD20 expressing B cells, respectively [4]. Furthermore, several lines of evidence suggest that the T cell compartment may be modulated as well, including reduction of antigen-presenting cells to pathogenic auto-reactive T cells and modulation of the regulatory T cell compartment [4]. Whereas transient depletion of peripheral B cells is common, Rituximab generally does not result in a decrease in immunoglobulin levels [4], a property that has been hypothesized to be attributable to the presence of long-lived plasma cells [33]. However, low immunoglobulin levels can occur, and in rare cases Rituximab may result in low numbers of isotype-switched memory B cells [31]. In a study involving Rituximab treatment of patients with rheumatoid arthritis, the incidence of decreased IgM was 40% whereas low IgG was seen in 3-6% of patients following the 5th cycle of Rituximab [34]. In addition rare cases have been published describing profound hypogammaglobulinemia seven years after treatment for indolent lymphoma [35] as well as hypogammaglobulinemia with selective delayed recovery in memory B cells after adjuvant Rituximab treatment for NHL [36].

### Previous cases of hypogammaglobulinemia associated with Rituximab treatment

Even if Rituximab only infrequently affects immunoglobulin levels, a central question is whether Rituximab may aggravate pre-existing CVID. Given that both CVID and Rituximab treatment result in loss of memory B cells and possibly affect the T cell population as well, it may be difficult to distinguish between the different origins of hypogammaglobulinemia. This issue was addressed by Diwakar et al., who described two patients presenting with aggravated immunodeficiency and bacterial infection requiring immunoglobulin infusions several months after Rituximab treatment for ITP [37], and therefore a very similar scenario to our case. This led the authors to suggest that Rituximab may have accelerated the presentation of immunodeficiency, since these patients had no notable infections prior to treatment but both experienced significant clinical deterioration, one of which was fatal, shortly after receiving Rituximab [37]. In addition, one report describing persistent pan-hypogammaglobulinemia after Rituximab treatment for post-transplant EBV-associated AIHA may also represent Rituximab-induced aggravation of CVID [38].

So should Rituximab always be avoided in patients with ITP, in whom CVID has been diagnosed or is suspected? Several reports suggest that this may not be the case [39-42]. In the first case, a 34-year-old male with CVID complicated by inflammatory bowel disease, hepatic hyperplasia, splenomegaly, and portal hypertension, obtained a partial response for at least 11 months, and the authors were the first to suggest that treatment with Rituximab might be an option for patients with CVID and ITP refractory to other treatments (or for those in whom splenectomy is contraindicated) [39]. Al-Ahmad et al. described the successful use of Rituximab in a patient with refractory ITP and CVID [40]. This patient, a 36-year-old female with associated lymphadenopathy, EBV vireamia, and bronchiectatic changes received Rituximab for ITP and experienced a good response, even though she had a well-recognized CVID. In the next case concerning a 19-year-old woman with CVID complicated by granulomatous disease, neutropenia, and refractory ITP, a full response to Rituximab treatment was achieved with effect at 12 months in combination with low-dose steroid [41]. Finally, El-Shanawany et al. described a case of a 65-year-old woman with CVID and severe recurrent thrombocytopenia despite treatment with high-dose IVIG and splenectomy, who had an excellent response to Rituximab therapy with disappearance of anti-platelet antibodies as reported 12 months after Rituximab [42]. However, in none of these cases did hypogammaglobulinemia occur as a direct side effect to Rituximab, as these patients were all diagnosed with CVID prior to Rituximab and, notably, all of these patients received IVIG concomitantly with and after Rituximab treatment. In line with these case reports Gobert et al. described the results of a multicenter retrospective study concerning the use of Rituximab in 33 patients with CVID-associated immune cytopenias and found that Rituximab was associated with a subsequent decrease in residual IgG in individuals not receiving IgG replacement therapy [24]. Furthermore, 24% of individuals developed severe infections after Rituximab treatment, and this was more frequently observed in those not receiving IgG replacement therapy. One splenectomized adult, who did not receive IgG replacement, died from *S. pneumoniae* pneumonia with sepsis 4 months after Rituximab treatment, underscoring the importance of addressing the potential development of severe hypogammaglobulinemia in patients with CVID treated with Rituximab.

### Pathogenesis of the development of hypogammaglobulinemia in the presented patient

We initially considered whether this patient’s hypogammaglobulinemia might be a direct consequence of Rituximab treatment, which he had received about 21 months prior to his presentation with pneumococcal meningitis. However, based on his past medical history, which included treatment resistant ITP, splenomegaly, persistent lymphadenopathy, chronic gastrointestinal complaints, insufficient antibody responses to pneumococcal vaccination, and decreased IgA levels, we speculated that his ITP might rather be an autoimmune manifestation of an underlying CVID. This hypothesis was supported by the immunological characterization demonstrating a very low fraction of isotype-switched memory B cells (IgM- IgD- CD27+) in agreement with a diagnosis of CVID Freiburg class Ia. However, it should be noted that decreased numbers of isotype-switched memory B cells may rarely be secondary to Rituximab [31]. Although we demonstrated neither TACI/ICOS mutations nor the absence of somatic hypermutation, CVID is a highly heterogeneous entity and these defects are only a few among several possible genetic abnormalities, which may be present in CVID. Based on the current knowledge on immunological effects of Rituximab, we do not believe that the concept of hypogammaglobulinemia arising as a direct effect of Rituximab sufficiently explains the case presented here. However, since this patient’s hypogammaglobulinemia became manifest and the frequency of infections increased following Rituximab infusions, it is possible that Rituximab was the factor that unmasked the full phenotype of an underlying CVID. In fact it was only after Rituximab treatment that he fulfilled the criteria defining CVID, in particular with respect to decreased IgG levels [2]. Importantly, he did not have any condition that excludes the diagnosis of CVID. It seems that the immunodeficiency became more dominant than the autoreactivity (ITP), both present in severe cases of CVID, following Rituximab treatment.

Due to the raised fraction of CD3+ TCRαβ + CD4- CD8- double negative T cells in peripheral blood, the possibility of autoimmune lymphoproliferative syndrome (ALPS) was also considered. ALPS is characterized by benign lymphoproliferative disease and autoimmune cytopenia with a functional defect of T cells in FAS-induced apoptosis/CD95 signaling and therefore has considerable clinical and immunological overlap with CVID [43]. However, in contrast to the case presented here, ALPS is generally characterized by hypergammaglobulinemia, although a minority of patients may present with hypogammaglobulinemia [44]. ALPS patients also display a distinct pattern of biomarkers, including elevated soluble FasL and IL-10, which we did not find, and have a less pronounced decrease in isotype-switched memory B cells than CVID patients. Finally, we cannot exclude a possible minor effect of the patient’s splenectomy at age 11 on the distribution of lymphocyte subpopulations measured by flow cytometry on peripheral blood [45], although we do not find it likely to account for the significant abnormalities, including absence of isotype-switched memory B cells.

## Conclusion

Taken together, we believe that the scenario that most precisely describes the clinical picture and immunological status in this patient is a diagnosis of underlying CVID, possibly aggravated by Rituximab. We recommend that all patients with ITP/AIHA should have their immunoglobulin levels measured repeatedly and be tested for CVID. Awareness of the association of CVID with autoimmune cytopenias is particularly important, since ITP/AIHA may often be diagnosed before the presence of hypogammaglobulinemia and thus be the presenting manifestation of CVID. This important point has been previously evidenced by several studies [13,46] and is also in line with the conclusions of a recent article discussing the same issue in patients with autoimmune cytopenias and underlying CVID or ALPS [47]. The case presented here together with other reports clearly illustrate that individuals with disturbed humoral immunity may be particularly sensitive to Rituximab [28]. In consideration of the well-established relationship between CVID and ITP, we recommend that humoral immunity, i.e. immunoglobulin levels, be examined before and after initiating Rituximab therapy. Rituximab treatment of patients with CVID and ITP does not seem to be contraindicated but should at least be accompanied by immunoglobulin replacement therapy during and after treatment [28,37]. In the future, it will be interesting to obtain further knowledge on whether Rituximab can be used effectively and safely in patients with CVID and ITP and to further explore the immunological effects of this potent therapeutic antibody.

## Consent

Written informed consent was obtained from the patient for publication of this Case report and any accompanying images. A copy of the written consent is available for review by the Editor of this journal.

## Abbreviations

AIHA: Autoimmune hemolytic anemia; B-CLL: B- cell chronic lymphocytic leukemia; CVID: Common variable immunodeficiency; ITP: Immune thrombocytopenic purpura.

## Competing interests

The authors declare that they have no competing interests.

## Authors’ contributions

THM, CSL, and PH cared for the patient, did the literature search and were responsible for the conclusions and writing of the final version of the manuscript. THM drafted the first version of the manuscript and together with CSL, PH, MH, and JMBJ organized and planned the immunological evaluation. JMBJ prepared Figure 1 and Additional file 1: Table S1. CCP and MH prepared Additional file 2: Figure S1. JMBJ, MSJ, CCP, MH, and CN performed the immunological analyses. KHG was the patient and provided details on his past medical history. THM and JMBJ were responsible for revision of the manuscript. All authors read and approved the final manuscript.

## Pre-publication history

The pre-publication history for this paper can be accessed here:

http://www.biomedcentral.com/2052-1839/13/4/prepub

## Supplementary Material

Additional file 1: Table S1 T and B cell subsets. Relative concentrations of T and B cell subsets are shown. Total T and B cell concentrations were as follows: January 2011: 1300 x 10^6^ /L and 285 x 10^6^ /L, respectively; and June 2011: 2812 x 10^6^ /L and 1089 x 10^6^ /L). ND: Not done. NA: Not available. Click here for file

Additional file 2: Figure S1 Flow cytometry plots of the bone marrow. A) Forward- and side scatter plot of the bone marrow, crude gate excluding dead cells and granulocytes. B) Doublet exclusion in a forward scatter area versus forward scatter height plot. C) CD19 APC versus CD20 FITC. Click here for file
