# Supplementary material for: Common variable immunodeficiency unmasked by treatment of immune thrombocytopenic purpura with Rituximab
Source: BMC Hematol. 2013 Apr 11;13:4. doi: 10.1186/2052-1839-13-4 (PMC3776283; doi:10.1186/2052-1839-13-4)
Supplement: Additional file 1: Table S1 — T and B cell subsets. Relative concentrations of T and B cell subsets are shown. Total T and B cell concentrations were as follows: January 2011: 1300 x 106 /L and 285 x 106 /L, respectively; and June 2011: 2812 x 106 /L and 1089 x 106 /L). ND: Not done. NA: Not available. [file 2052-1839-13-4-S1.pdf]

			Percentage of parent population (total numbers x 10^6/L)		
**Cell type**	**Subset**	**Lymphocyte phenotype**	**January 2011**	**June 2011**	**Reference**
**T cells**	All	CD3+CD4+CD8-	55	54	[43;54]
	Naïve (1)	CD4+CD45RA+CD45RO-	22	ND	[22;67]
	Naïve (2)	CD3+CD4+CD8-CCR7+CD27+CD45RA+	28	21	NA
	Memory (1)	CD4+CD45RA-CD45RO+	76	ND	[35;82]
	Central memory	CD3+CD4+CD8-CCR7+CD27+CD45RA-	59	65	NA
	Effector memory	CD3+CD4+CD8-CCR7-CD27+CD45RA-	9.2	9.0	NA
	Effector memory	CD3+CD4+CD8-CCR7-CD27-CD45RA(-/+)	2.9	3.3	NA
	All	CD3+CD4-CD8+	30	32	[28;37]
	Naïve (1)	CD8+CD45RA+CD45RO-	57	ND	[31;78]
	Naïve (2)	CD3+CD4-CD8+CCR7+CD27+CD45RA+	31	32	NA
	Memory (1)	CD8+CD45RA-CD45RO+	36	ND	[24;69]
	Central memory	CD3+CD4-CD8+CCR7+CD27+CD45RA-	4.1	5.8	NA
	Effector memory	CD3+CD4-CD8+CCR7-CD27+CD45RA(-/+)	47	41	NA
	Effector memory	CD3+CD4-CD8+CCR7-CD27-CD45RA(-/+)	19	21	NA
	Double negative	CD3+CD4-CD8-	15	13	NA
	Double positive	CD3+CD4+CD8+	0.4	0.3	NA
**B cells**	Marginal zone-like	CD19+/CD20+IgM+IgD+CD38-CD27+CD3-	3.5 (10.0)	5.0 (54.5)	[7.4;33]
	Transitional	CD19+/CD20+IgM+IgD+CD38+CD27-CD3-	20 (57)	12 (130.7)	[0.6;3.4]
	Naïve	CD19+CD20+IgM+IgD+CD38-CD27-CD3-	75 (213.8)	80 (871.2)	[43;82]
	Isotype-switched, memory	CD19+/CD20+IgM-IgD-CD38-CD27+CD3-	0.1 (0.3)	0.1 (1.1)	[6.5;29]
	Plasmablast	CD19+/CD20-IgM(-/dim)IgD-CD38+CD27+	0.0 (0)	0.0 (0)	[0.4;3.6]
	CD21low	CD19+/CD20+CD38-CD21dim	24 (68.4)	20 (217.8)	[0.9;7.6]

Supplementary Table 1. T and B cell subsets. Relative concentrations of T and B cell subsets are shown. Total T- and B-cell concentrations were as follows: January 2011: 1300 x 10^6/L and 285 x 10^6/L, respectively; and June 2011: 2812 x 10^6 /L and 1089 x 10^6 /L). ND: Not done. NA: Not available.
