# Supplementary material for: Common variable immunodeficiency unmasked by treatment of immune thrombocytopenic purpura with Rituximab
Source: BMC Hematol. 2013 Apr 11;13:4. doi: 10.1186/2052-1839-13-4 (PMC3776283; doi:10.1186/2052-1839-13-4)
Supplement: Additional file 2: Figure S1 — Flow cytometry plots of the bone marrow. A) Forward- and side scatter plot of the bone marrow, crude gate excluding dead cells and granulocytes. B) Doublet exclusion in a forward scatter area versus forward scatter height plot. C) CD19 APC versus CD20 FITC. [file 2052-1839-13-4-S2.pdf]

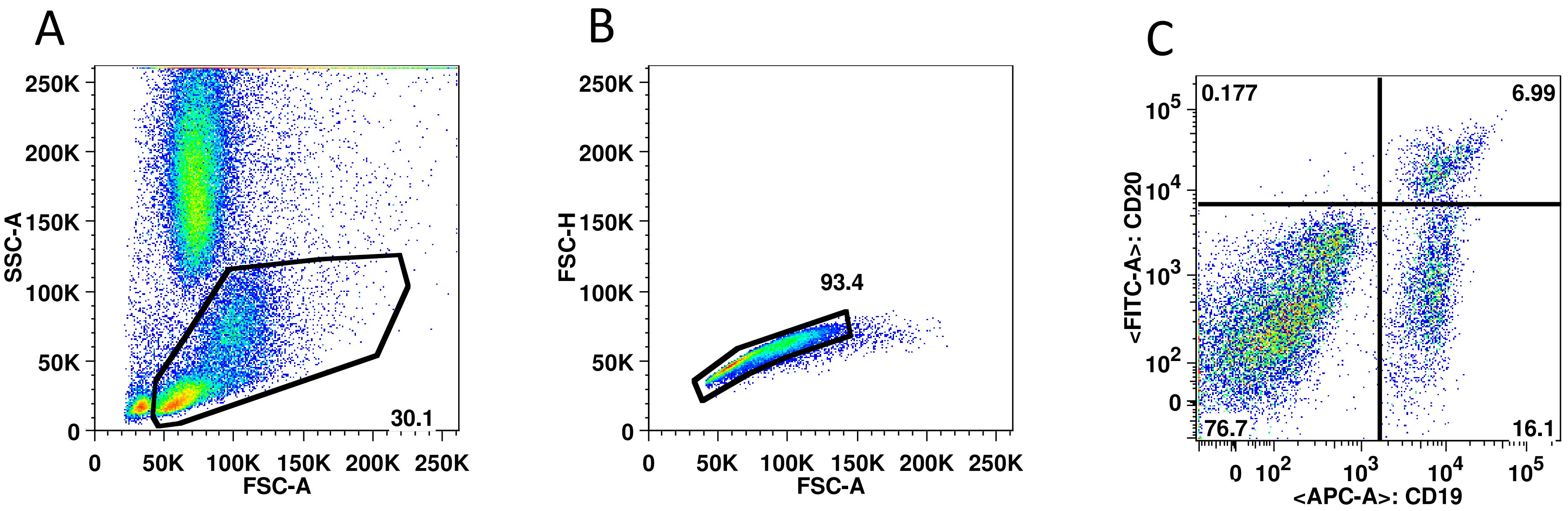

Supplementary Fig. 1. Flow cytometry plots of the bone marrow. A) Forward- and side scatter plot of the bone marrow, crude gate excluding dead cells and granulocytes. B) Doublet exclusion in a forward scatter area versus forward scatter height plot. C) CD19 APC versus CD20 FITC.
